# Rescuing Suboptimal Patient-Reported Outcome Instrument Data in Clinical Trials: A New Strategy

**DOI:** 10.3390/healthcare6010027

**Published:** 2018-03-20

**Authors:** Chengwu Yang, Kent E. Vrana

**Affiliations:** 1 Departments of Epidemiology and Health Promotion, College of Dentistry, New York University, New York, NY 10010, USA; 2 Department of Pharmacology, College of Medicine, The Pennsylvania State University, Hershey, PA 17033, USA; kev10@psu.edu

**Keywords:** patient-reported outcome (PRO), clinical trials, instrument, measurement, scale, psychometrics, clinimetrics, Parkinson’s disease, RBANS, factor analysis, placebo effect

## Abstract

*Background*: Psychometric instruments such as the Repeated Battery for the Assessment of Neuropsychological Status (RBANS) are commonly used under conditions for which they were not developed or validated. They may then generate troublesome data that could conceal potential findings. *Methods*: Based on a previously published refinement of the RBANS, we reanalyzed the data on 303 patients from two National Institutes of Health (NIH) trails in Parkinson’s disease and contrasted the results using the original versus refined scores. *Results*: Findings from the original RBANS scores were inconsistent; however, use of the refined scores produced potential findings that were in agreement with independent reports. *Conclusion*: This study demonstrates that, for negative trials using instrument scores as primary outcomes, it is possible to rescue potential findings. The key to this new strategy is to validate and refine the instrument for the specific disease and conditions under study and then to reanalyze the data. This study offers a demonstration of this new strategy for general approaches.

## 1. Introduction

Since few health-related psychometric instruments have been “professionally developed” [1], it is common to see suboptimal instrument data in clinical trials. An example was the use of the Repeatable Battery for the Assessment of Neuropsychological Status (RBANS) [2] in two National Institutes of Health (NIH) Exploratory Trials in Parkinson’s disease (NET-PD) [3,4]. The RBANS has been popular since its initial publication. According to Web of Science (Thomson Reuters; accessed 12 December 2017), the initial description of RBANS has been cited 472 times. Moreover, it has been translated and used in many other countries such as China [5], Japan [6], and Italy [7]. Its popularity may relate to its brevity. However, the original factor structure of RBANS was theory-driven [2], while multiple subsequent empirical studies have identified optimal factor structures that differ from the original (e.g., [8,9,10,11,12,13,14]). This has engendered significant concerns about the validity of the universal use of RBANS to assess cognitive function.

Two NET-PD trials tested four drugs for the treatment of movement impairment in PD: Creatine and Minocycline in FS1, and CoQ10 and GPI1485 in FS-TOO, indicating that Creatine and Minocycline might be promising [3,4]. The RBANS was used to assess cognition as a secondary outcome. Because we previously demonstrated that the original RBANS had not been validated for PD, it is not surprising that the RBANS assessments produced equivocal results [13]. Yet, we believe that better use of these problematic, but expensive, data is of critical and practical importance. We therefore set out to reanalyze the RBANS data from the two NET-PD trials based on the refined factor structure from our previous study [13], and then to contrast the results with those based on the original factor structure, in hope of rescuing potential findings.

## 2. Materials and Methods

### 2.1. Patients

The two NET-PD trials recruited 858 early untreated PD patients from 42 sites in North America, and randomized 413 participants into six arms. Details can be found in earlier publications [3,4]. In total, 339 finished the 12-month follow-up visit. RBANS data were collected at the baseline and 12-month follow-up visit. After the deletion of patients with missing values for the RBANS data and outliers as detected by the Malhanobis distance, 383 and 315 patients remained at baseline and follow-up. Since the change from baseline to follow-up in RBANS scores will be used as the primary outcome, only patients with complete RBANS data at both baseline and follow-up were included in the final analysis of this study. Therefore, 303 patients with complete RBANS data at both baseline and follow-up were analyzed.

### 2.2. The RBANS

**Figure 1:**
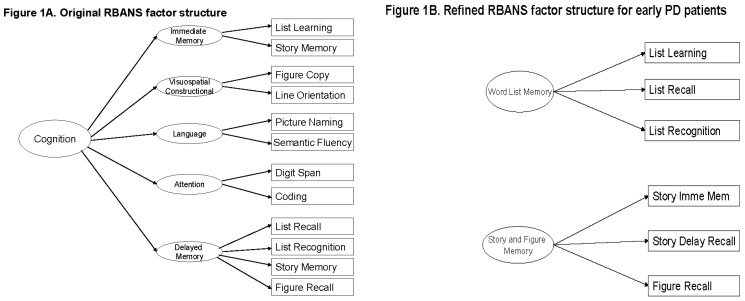
Factor structure of the RBANS: Original vs. refined for early PD patients.

The original RBANS has 12 items and offers scores on five cognitive domains [2]. Each of the first four domains is measured by two items, while the last domain is measured by four. In addition, the RBANS offers a total score based on the five domain scores (Figure 1A). However, these six original RBANS scores are neither valid nor reliable in the two NET-PD trials [13].

Following psychometric analysis, we created a new instrument that retained half of the assessment items from the original RBANS and reorganized them into two domains [13]. The refined RBANS [13,15] for these trials has six items and offers scores on two new cognitive domains: word list memory (WLM) and story and figure memory (SFM), measured by three items each (Figure 1B). Specifically, six of the original RBANS items were excluded because they had low correlation with each other and the remaining six items [13]. The refined RBANS is valid and reliable for these specific patients, and makes clinical sense [13]. However, a unified total RBANS score based on these two domains was not supported by a two-level factor structure [13].

### 2.3. Statistical Analysis

Data from the two trials were analyzed separately and in parallel. The normality of each variable was checked in order to choose appropriate analysis approaches. Chi-square tests were applied to categorical data, and analysis of variance (ANOVA) or Kruskal-Wallis tests were applied to continuous data. Demographic and disease characteristics of the participants at baseline were summarized and compared across the three arms within each of the two trials. Preliminary comparisons of change from baseline on cognitive abilities as measured by the original and refined RBANS, within each of the two trials, were implemented. In comparing the original RBANS with the refined RBANS, we focused on providing consistent changes in cognitive scores. Comparing *p*-values would be flawed given the lack of power in these historical data (inadequate sample size) for assessing these secondary outcome measures [3,4]. Post hoc power analysis and sample size estimation were implemented to address the lack of power and small sample size issues. SPSS (Version 23, IBM Corp., Armonk, NY, USA) and SAS (Version 9.4, SAS Institute Inc., Cary, NC, USA) were used for data preparation and analysis. G*Power (Version 3.1.9.2, University of Düsseldorf, Düsseldorf, Germany) [16,17]) was used for post hoc power analysis and sample size estimation.

## 3. Results

Demographic and disease characteristics at baseline are summarized in Table 1.

No statistically significant difference was detected at any of these characteristics among the two treatment groups and one placebo group within each of the trials, and characteristics are very similar within each of the two trials.

Preliminary comparisons of change from baseline on cognitive abilities are summarized in Table 2.

When cognition is measured by the original six RBANS scores, there is no consistent trend over time. Out of the 36 changes, 17 (47.2%) are increased, 19 (52.8%) are decreased. Moreover, none of the six groups show consistency in the direction of change among the original six RBANS scores. For example, in the Creatine group, while four original RBANS scores show increases, the other two show decreases. In contrast, when cognition is measured by the two refined RBANS scores, the trend over time is much more consistent. Out of the 12 changes on refined RBANS scores, 10 (83.3%) show increases, and only two show a decrease, but both within one unit (−0.12, −0.83). None of the differences among groups, in change from baseline on either of the RBANS scores, is statistically significant (*p* > 0.05), because the study was underpowered. Clearly, however, the refined RBANS provides much more consistent data trends, and will therefore give greater insight into treatment outcomes.

Post hoc power analysis indicated that, in order to have 80% power to detect the difference shown on WLM between Creatine and Placebo (3.00 vs. 1.25, Cohen’s d = 0.30 [19]), 178 patients per group would be needed. Given the current sample size of 50 per group, the statistical power to detect this difference was 31%. Since the effect size of Creatine on WLM was the largest, post hoc analysis on others would result in a larger required sample size or would show lower power at the current sample size.

## 4. Discussion

Given that suboptimal instrument data are commonly utilized in clinical trials, it is of great practical importance to better utilize these troublesome and expensive data in hope of rescuing important potential findings. This study offers a template for rescuing efforts through reanalyzing existing suboptimal instrument data. Findings from the two NET-PD trials that employed the unified Parkinson’s disease rating scale (UPDRS) scores as the primary outcome indicated that two (Creatine, Minocycline) of the four tested drugs may be beneficial for PD patients [3], while the other two (CoQ10, GPI1485) may not [4]. Results from the present study indicate that, while using the original six RBANS scores showed no benefit of the treatments on cognitive ability as a secondary outcome, the use of the two refined RBANS scores may have produced a positive outcome had the sample sizes been larger (Table 2).

The inconsistencies in the trend among the original six RBANS scores are very troublesome. It is not easy to explain why a drug can help improve some cognitive abilities while impairing others (Table 2). This observation strengthens the conclusion that the RBANS was neither valid nor reliable in the two NET-PD trials studied here [13]. In contrast, the refined RBANS scores offer much more consistency in the trend, albeit in the absence of statistical significance. Most of the changes from baseline are increasing, indicating that the treatment is increasing each of the cognitive abilities. The increased outcomes in cognitive assessments offer more practical support for the validity and reliability of the refined RBANS in both trials [13].

Another advantage of the refined versus the original RBANS is indicated by the big differences in the standard deviations (SD) of the changes from baseline (Table 2). When using the original RBANS, the SDs are huge (e.g., for Creatine, Att has a mean of 0.65 and an SD of 13.17). However, after refinement, the SDs dropped substantially (e.g., for Creatine, WLM has a mean of 3.00 and an SD of 5.52.

Placebo effect [20] on the two refined RBANS scores is evidenced by the three increases in the two placebo groups. Participating in a clinical trial and receiving some kind of treatment may help patients feel better and can improve their cognition. However, these placebo effects are smaller than potential true treatment effects.

Lack of power due to the small sample size is the primary reason for potentially physiologically important, but statistically negative results. Had the sample size been large enough, these results should also be statistically significant. Future studies with appropriate sample size are therefore warranted.

Clearly, there are important limitations in this analysis related to statistical power. Take, for example, the observed differences on WLM between Creatine and Placebo in FS1 (details in Table 2). The observed difference was 3.00 for Creatine and 1.25 for Placebo, with Cohen’s d as 0.30, which was between “small” and “medium” [19]. That is to say, the difference was clearly “clinically significant”. However, due to the small sample size (46 in Creatine, 53 in Placebo, 99 total), the *p*-value was 0.15, and the difference was “statistically insignificant”. This is a typical scenario when a study finding is “clinically significant”, but “statistically insignificant”, since the sample size is not big enough. However, clinical significance should be an important deciding factor for medical studies, not simply statistical significance, because “*p*-values do not measure evidence” [20] (p. 619). In addition, recent reports have re-emphasized the severe issue of *p*-driven research (e.g., [21]), including the American Statistical Association (ASA) statement on *p*-values [22]. What the present studies emphasize, however, is that a properly designed and validated instrument, combined with an appropriate sample size, can provide both clinical and statistical significance.

In clinical trials that use psychometric instruments, it is critical to validate or even refine the instruments for data collected before any formal statistical analysis. This is because most instruments are “not professionally developed” [1], and instrument validation is an “ongoing process” [23]. No instrument should be claimed to be “already validated”; rather, assessment instruments should be validated for the disease and population under study. Another good example appears a recent review on oral health-related quality of life (OHRQoL) instruments [24]. Other recent literature re-emphasizes the importance of sound psychometric properties of an instrument [25,26,27].

For negative trials that used instrument scores as primary outcomes, the present findings offer a path to rescuing potential findings: validating and refining the instruments and then reanalyzing the data based on the refined instrument scores. Our study offers a demonstration of the new strategy for this type of promising effort.

## 5. Conclusions

This study demonstrates that, for negative trials using instrument scores as primary outcomes, it is possible to rescue potential findings. The key to this new strategy is to validate and refine the instrument for the specific disease and conditions under study and then to reanalyze the data. This study offers a demonstration of this new strategy for general approaches.

## Figures and Tables

**Table 1:** Demographic and disease characteristics of participants at baseline.

**FS1 (n = 151)**		**1. Creatine (n = 46)**	**2. Minocycline (n = 52)**	**3. Placebo (n = 53)**	***p*-Value**
Age	Mean ± SD	61.0 ± 10.8	64.1 ± 10.5	60.7 ± 9.7	0.11
Education	Mean ± SD	15.4 ± 3.5	15.0 ± 2.9	15.2 ± 2.9	0.95
Gender	Male	32 (69.6%)	28 (53.9%)	34 (64.2%)	0.26
	Female	14 (30.4%)	24 (46.1%)	19 (35.8%)	
Race	White	43 (93.5%)	51 (98.1%)	49 (92.5%)	0.44
	Others *	3 (6.5%)	1 (1.9%)	4 (7.5%)	
Ethnicity	Not Hispanic or Latino	45 (97.8%)	51 (98.1%)	51 (96.2%)	0.99
	Others †	1 (2.2%)	1 (1.9%)	2 (3.8%)	
UPDRS Total Score (Parts I–III)	Mean ± SD	22.8 ± 8.9	23.7 ± 9.2	23.0 ± 10.3	0.78
Schwab and England Activities of Daily Living scale	Mean ± SD	92.9 ± 5.1	92.5 ± 6.4	94.5 ± 4.6	0.17
Tremor Dominant ‡	Yes	21 (45.7%)	25 (48.1%)	33 (62.3%)	0.19
	No	25 (54.3%)	27 (51.9%)	20 (37.7%)	
Hoehn and Yahr Staging	0	0	0	0	0.46
	1	27 (58.7%)	24 (46.1%)	27 (50.9%)	
	2	19 (41.3%)	28 (53.9%)	26 (49.1%)	
	3	0	0	0	
**FS-TOO (n = 152)**		**1. CoQ10 (n = 50)**	**2. Minocycline (n = 50)**	**3. Placebo (n = 52)**	***p*-Value**
Age	Mean ± SD	61.1 ± 9.2	61.4 ± 10.9	61.4 ± 9.2	0.90
Education	Mean ± SD	15.6 ± 3.2	15.4 ± 3.0	15.4 ± 2.7	0.97
Gender	Male	31 (62.0%)	32 (64.0%)	38 (73.1%)	0.45
	Female	19 (38.0%)	18 (36.0%)	14 (26.9%)	
Race	White	47 (94.0%)	49 (98.0%)	47 (90.4%)	0.30
	Others *	3 (6.0%)	1 (2.0%)	5 (9.6%)	
Ethnicity	Not Hispanic or Latino	50 (100.0%)	48 (96.0%)	52 (100.0%)	0.21
	Others †	0	2 (4.0%)	0	
UPDRS Total Score (Parts I–III)	Mean ± SD	22.0 ± 9.6	20.8 ± 8.8	22.2 ± 9.1	0.67
Schwab and England Activities of Daily Living scale	Mean ± SD	92.9 ± 5.7	93.8 ± 4.4	93.1 ± 4.9	0.78
Tremor Dominant ‡	Yes	26 (52.0%)	31 (62.0%)	27 (51.9%)	0.50
	No	24 (48.0%)	19 (38.0%)	25 (48.1%)	
Hoehn and Yahr Staging	0	1 (2.0%)	0	0	0.96
	1	27 (54.0%)	28 (56.0%)	30 (57.7%)	
	2	21 (42.0%)	22 (44.0%)	22 (42.3%)	
	3	1 (2.0%)	0	0	

* Other races include Indian, Alaska Native, Asian, Black/African American, more than one race, and unknown or not reported. † Other ethnicities include Hispanic or Latino, and unknown or not reported. ‡ A PD patient was determined to be tremor dominant or not by using the method described by Jankovic et al. (1990) [18]. UPDRS: unified Parkinson's disease rating scale.

**Table 2:** Preliminary comparisons of change from baseline on cognitive abilities among treatment groups using the Repeated Battery for the Assessment of Neuropsychological Status (RBANS) scores: original vs. refined.

**FS1 (n = 151)**	**RBANS Scores**		**Creatine (n = 46)**		**Minocycline (n = 52)**		**Placebo (n = 53)**		***p*-Value**
			**Mean**	**SD**	**Mean**	**SD**	**Mean**	**SD**	
	Original	IM	5.93	12.79	4.63	12.18	3.77	11.95	0.86
		VC	−2.91	14.43	−7.29	18.37	−2.28	17.39	0.34
		La	−1.22	10.52	−2.94	9.33	0.53	9.34	0.09
		Att	0.63	13.17	−1.38	14.43	−0.94	12.48	0.87
		DM	2.09	13.49	−0.52	11.45	−2.28	14.28	0.26
		Total	1.39	9.77	−2.81	10.04	−0.94	9.81	0.14
	Refined	WLM	3.00	5.52	0.75	5.55	1.25	5.95	0.15
		SFM	1.11	7.91	0.87	7.36	0.11	6.25	0.95
**FS-TOO (n = 152)**	**RBANS Score**		**CoQ10 (n = 50)**		**GPI1485 (n = 50)**		**Placebo (n = 52)**		***p*-Value**
			**Mean**	**SD**	**Mean**	**SD**	**Mean**	**SD**	
	Original	IM	0.60	10.24	0.18	10.41	0.19	14.98	0.82
		VC	−0.96	14.45	−5.72	16.90	−1.94	14.76	0.50
		La	1.44	9.55	−0.16	8.11	−1.60	8.38	0.13
		Att	−0.02	13.09	0.02	12.90	1.65	13.69	0.71
		DM	4.78	14.23	1.18	20.24	0.37	14.63	0.51
		Total	2.14	9.82	−1.16	10.43	−0.52	10.87	0.24
	Refined	WLM	0.10	4.71	0.60	4.92	0.52	6.12	0.80
		SFM	1.52	5.66	−0.12	6.86	−0.83	5.36	0.13

None of the pairwise comparisons of the refined RBANS scores was statistically significant (*p*-value not shown), mainly due to the small sample size; IM: Immediate Memory; VC: Visuospatial/Constructional; La: Language; Att: Attention; DM: Delayed Memory; WLM: Word List Memory; SFM: Story and Figure Memory [13].
